# Multi-National Banknote Classification Based on Visible-light Line Sensor and Convolutional Neural Network

**DOI:** 10.3390/s17071595

**Published:** 2017-07-08

**Authors:** Tuyen Danh Pham, Dong Eun Lee, Kang Ryoung Park

**Affiliations:** Division of Electronics and Electrical Engineering, Dongguk University, 30 Pildong-ro 1-gil, Jung-gu, Seoul 100-715, Korea; phamdanhtuyen@gmail.com (T.D.P.); exexzz@naver.com (D.E.L.)

**Keywords:** multi-national banknote classification, visible-light banknote images, one-dimensional line sensor, convolutional neural network

## Abstract

Automatic recognition of banknotes is applied in payment facilities, such as automated teller machines (ATMs) and banknote counters. Besides the popular approaches that focus on studying the methods applied to various individual types of currencies, there have been studies conducted on simultaneous classification of banknotes from multiple countries. However, their methods were conducted with limited numbers of banknote images, national currencies, and denominations. To address this issue, we propose a multi-national banknote classification method based on visible-light banknote images captured by a one-dimensional line sensor and classified by a convolutional neural network (CNN) considering the size information of each denomination. Experiments conducted on the combined banknote image database of six countries with 62 denominations gave a classification accuracy of 100%, and results show that our proposed algorithm outperforms previous methods.

## 1. Introduction

In practice, automated transaction facilities, such as automated teller machines (ATMs) and multifunctional counting machines, should be able to process various tasks, such as denomination determining, fitness classification, and counterfeit detection, with currencies from various countries and regions [1]. Regarding banknote recognition, one of the most popular approaches is based on an image processing method, which has been considered an effective solution [2,3]. In this method, a recognition system captures an input banknote by using visible light sensors and determines its denomination based on the classification of the input direction of banknote.

Many studies involved in the classification of banknotes from different countries conducted experiments on separated image datasets of each country’s banknote. The method proposed by Gai et al. [4] uses quaternion wavelet transform (QWT) and generalized Gaussian density (GGD) for feature extraction, and neural network (NN) for classification of banknote images from the United States (US), China, and Europe. In the method proposed by Pham et al. [5], currency of the US dollar (USD), South African rand (ZR), Angolan kwanza (AOA), and Malawian kwacha (MWK) are recognized by a K-means-based classifier with the features extracted by principal component analysis (PCA) of the discriminative regions on banknote images. Using color features of hue, saturation, and value (HSV) model, Bhurke et al. [6] proposed a Euclidian distance-based banknote recognition and built a graphical user interface (GUI) for displaying the results on the Indian rupee (INR), Australian dollar (AUD), Euro (EUR), Saudi Arabia riyal (SAR), and USD. There has also been research on USD, INR, and Korean won (KRW) from Kwon et al. [7], however, they focused on banknote fitness classification.

Additionally, there have been studies on simultaneous recognition of multiple currencies from various countries. An NN was used as the combined classifier for two different types of banknote, the Cyprus Pound and Turkish Lira, in the study of Khashman and Sekeroglu [8]. The two banknote types of USD and EUR are also recognized simultaneously in the method proposed by Rashid et al. [9]. In this study, they conducted comparative experiments using three classification techniques. These are the support vector machine (SVM), artificial neural network (ANN), and hidden Markov model (HMM). The multi-currency classification method proposed by Youn et al. [10] adopted multi-template correlation matching to determine the areas on banknote images used for recognition, such that they ensure high correlation among banknotes of the same types and poor correlation among those of different types. Rahman et al. [11] proposed a linear discriminant analysis (LDA)-based recognition method using an edge histogram descriptor (EHD) for classifying banknotes of 14 denominations from four types of currencies including USD, EUR, Bangladeshi taka (BDT), and INR. Another four types of currencies from Japan, Italia, Spain, and France were previously classified in the research of Takeda et al. [12]. In this multi-national currency recognition method, the NN was also used as the classifier for the banknote feature extracted by genetic algorithm (GA)-based optimized masks. A dataset consist of 150 denominations of banknotes from 23 countries were used for assessing the performance of HMM-based paper currency recognition proposed by Hassanpour and Farahabadi [13].

The NN has been used as the classifier in many previous studies because of its effectiveness in the solution of the multiclass classification in the banknote recognition problem [4,8,9,12,14,15,16,17,18,19]. The network model can be either learning vector quantization (LVQ)-based [14,15,16,17] or multi-layered perceptron (MLP)-based [4,8,9,12,18,19]. In these studies, features from banknote images are extracted by various methods, such as wavelet transform [4], pixel averaging [8], scale-invariant feature transform (SIFT) [9], symmetrical masks on banknote images [12,19], edge feature extraction [14,18], and PCA feature extraction [15,17]; and subsequently fed into the NN for determination of denomination or input direction. The recent banknote detection and recognition methods aiming to visual impaired users used speeded up robust feature (SURF) for feature extraction [20,21]. Because the banknotes in these studies were captured by camera mounted on sunglasses, the problems of rotation, scaling and illumination changes can be handled by the robustness of SURF for geometric and photometric variation as well as speed improvement [20].

Recently, convolutional neural network (CNN) algorithms have been emerging and playing an important role in the development of deep learning and artificial intelligent technology. Since the first introduction by LeCun et al. in the review research of handwritten character recognition [22,23], CNN has been adopted for solving various problems, such as image classification for the ImageNet large-scale visual recognition challenge (ILSVRC) contest [24,25], arrow-read marker [26], traffic sign recognition [27], and multi-sensor-based person recognition [28]. However, there is little previous research conducted on banknote recognition using CNN. Recently, Ke et al. proposed banknote image defect recognition using CNN [29], however, they focused only on ink dot recognition in a banknote image defect and did not specify which type of banknote was used in the experimental dataset. To overcome these shortcomings, we propose CNN-based recognition of banknote images captured by visible-light line sensors. Owing to the advantage of deep learning by a convolutional network, our proposed method is designed to simultaneously classify banknote from multiple countries. To reduce the complexity of the classifier, first, we perform the size pre-classification of banknote. The pre-classified banknote is subsequently normalized in size and input into the CNN classifier.

Compared to the previous studies, our proposed method is novel in the following:

(1) This is the first approach to adopt CNN for multi-national banknote classification. We performed intensive training on the CNN using a huge number of banknote images obtained through data augmentation based on the images of six national currencies having 62 denominations, which makes our method robust for a variety of banknote images.
(2) Considering the size characteristics of a conventional banknote image, we use a size normalized image whose width is larger than height for input to CNN. This is different from the previous methods of CNN-based object detection and recognition using the square-shaped input. In addition, in our method, the input banknote is captured on both sides, and we use score level fusion method to combine the CNN output scores of the front and back images to enhance recognition accuracy.
(3) Our recognition system can simultaneously classify banknotes from six national currencies: Chinese yuan (CNY), EUR, Japanese yen (JPY), KRW, Russian ruble (RUB), and USD. Because the input banknote image is recognized by denomination and direction, the number of classes is increased significantly. To reduce the complexity, we pre-classify the type of banknote by size, and adopt separated CNN classifiers for the size classes of the banknote in our system.
(4) We made our database of multi-national currencies and trained CNN model public such that other researchers can compare and evaluate its performance.

A summary comparison between our research and previous studies is given in Table 1. In Section 2, we present the details of the proposed multi-national banknote recognition method. Experimental results and conclusions drawn are presented in Section 3 and Section 4 of this paper, respectively.

## 2. Proposed Method 

### 2.1. Overview of the Proposed Method

**Figure 1:**
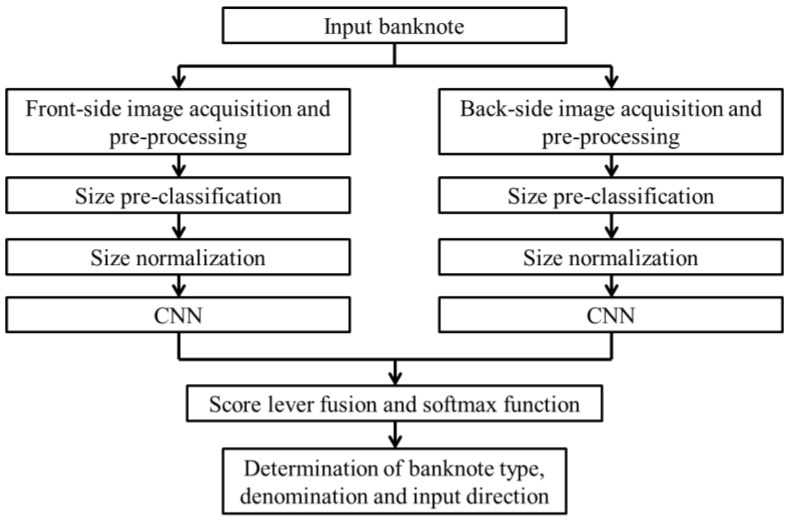
Overall flowchart of the proposed method.

The overall flowchart of our proposed banknote recognition method is shown in Figure 1. When a banknote is input to the system, the images on both sides of the banknote are captured and pre-processed. In the pre-processing step, the banknote region is segmented from the background and size pre-classification is conducted based on the segmented banknote image sizes.

Because the size of the input image to CNN should be the same, the segmented banknote image is resized to 115 × 51 pixels. The equally resized banknote image is fed into the pre-trained CNN. At this step, we use images from both sides of input banknote and combine by averaging the CNN output scores. Finally, the origin country, denomination, and input direction of the banknote is determined by means of a softmax function on the combined scores [30,31].

### 2.2. Banknote Image Acquisition and Pre-Processing

**Figure 2:**
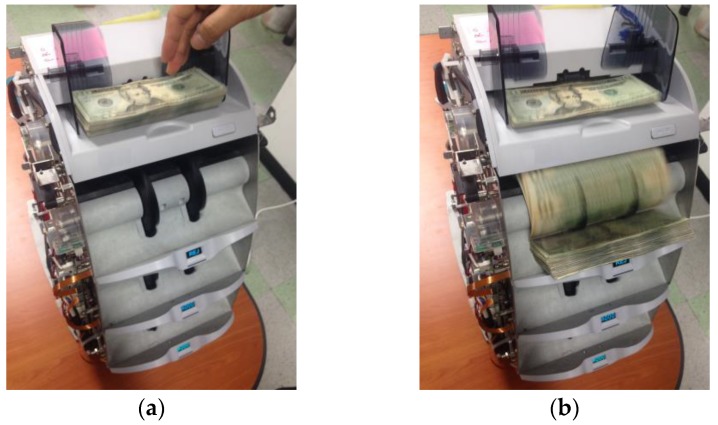
Examples of data acquisition of our research: (**a**) Input banknotes; (**b**) Acquisition of image data.

In this study, we used a commercial banknote counting machine with one-dimensional visible-light sensors for capturing the banknote images [32]. Because of size and cost limitations of the counting machine, a conventional two-dimensional (area) image sensor is not adopted. To obtain an entire image of a banknote while being rolled through the counting device, each one-dimensional (line) image of the banknote is captured by the line sensor successively at a high speed while being illuminated by a light-emitting diode (LED). The line sensor is triggered 464 times, and has a resolution of 1584 pixels. Consequently, by concatenating the captured line images, we can obtain a two-dimensional image of the input banknote with a resolution of 1584 × 464 pixels. Figure 2 shows our research set-up. As shown in Figure 2a, when we input the banknotes into the banknote-counting machine, the image data of each banknote can be automatically obtained as shown in Figure 2b.

**Figure 3:**
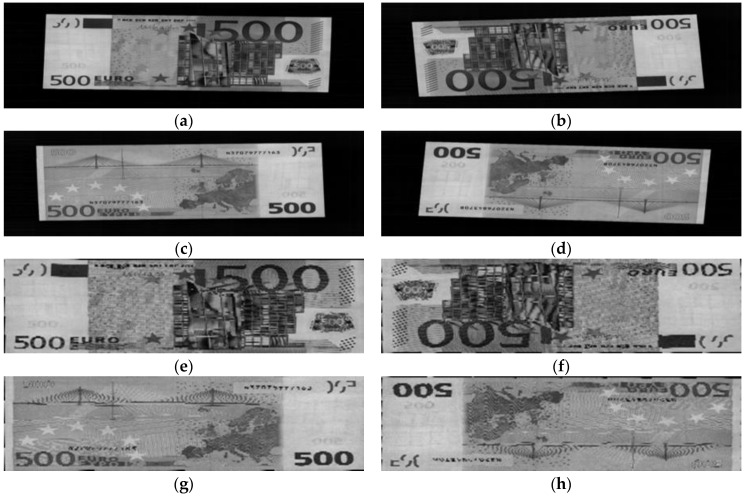
Examples of EUR banknote images and their corresponding banknote region segmented images in four input directions: (**a**) A direction; (**b**) B direction; (**c**) C direction; (**d**) D direction; (**e**–**h**) Corresponding banknote region segmented images from the original captured images in (**a**–**d**).

When using the contact image sensor, a banknote image can be captured in one of four possible directions: forward direction of obverse side, backward direction of obverse side, forward direction of reverse side, and backward direction of reverse side, denoted by A, B, C, and D directions, respectively. Examples of the four input directions of the EUR 500 banknote are shown in Figure 3. It can be seen from Figure 3a–d that the captured banknote image consists of both banknote region and surrounding background. By using a commercial corner detection algorithm built into the counting machine, we can segregate the banknote region from background area [5,33], and obtain the rough size information of the input banknote. This step also assists in fixing rotation and displacement problems when placing the banknote into the system. Examples of a segmented banknote region from an original captured image are also given in Figure 3.

### 2.3. Banknote Size Pre-Classification

**Figure 4:**
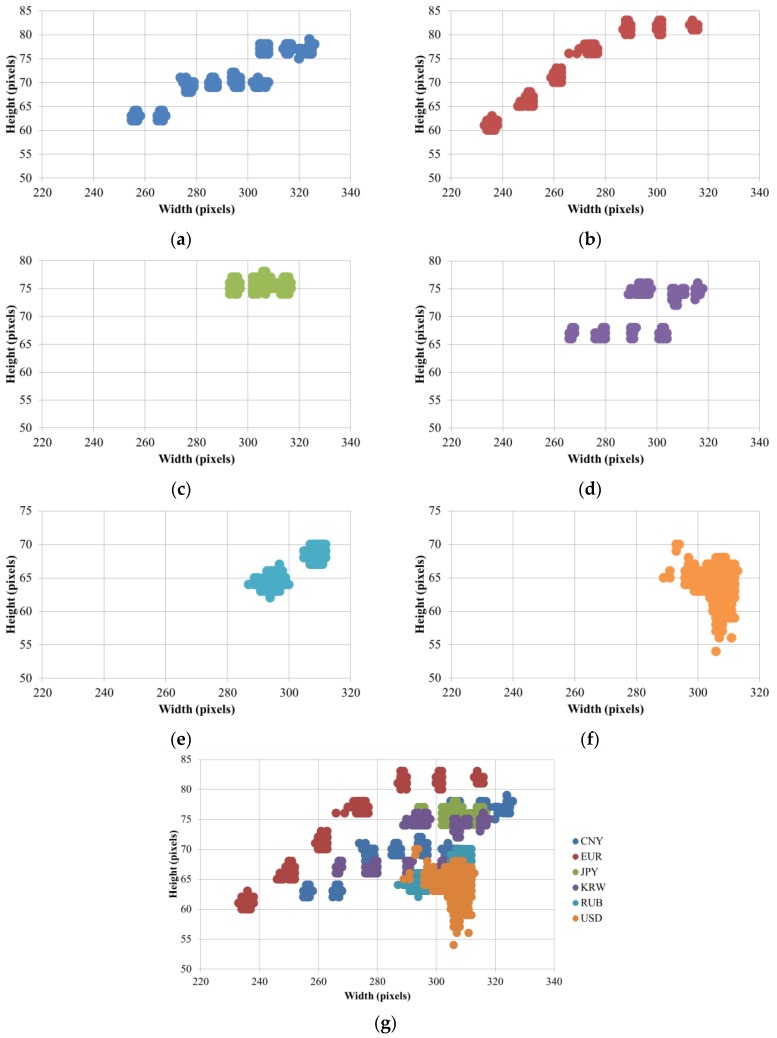
Banknote size distribution of: (**a**) CNY; (**b**) EUR; (**c**) JPY; (**d**) KRW; (**e**) RUB; (**f**) USD; and (**g**) all the above national currency papers.

Because certain banknotes of different countries and denominations have different sizes, in this step, we use size information obtained from banknote region segmented images for pre-classification. First, the size distribution of the banknote was examined via banknote region segmented images of the six national currency papers to be classified in this research, and shown in the scatter plots of heights and widths of these images in Figure 4.

Despite the variation in the capturing and segmenting processes, the difference among groups of banknotes that have similar sizes is still considerable compared to variation due to pre-processing in the same group. For instance, it can be seen from Figure 4 that EUR banknotes have seven size groups and are considerably separate from other banknote size distributions.

However, from Figure 4, we can see that there is overlap between size groups of different countries’ banknotes. Thus, when defining size classes for pre-classification, a group of banknotes can be included in different size classes, in considering the coincidence of the size among different types of banknotes. A detail definition of banknote size classes is given in Section 3 of this paper. For each size class, we build separated CNN models as classifiers for recognizing the origin nation, denomination, and input direction of the banknote belonging to a corresponding size range.

### 2.4. The Architecture of CNN

**Figure 5:**
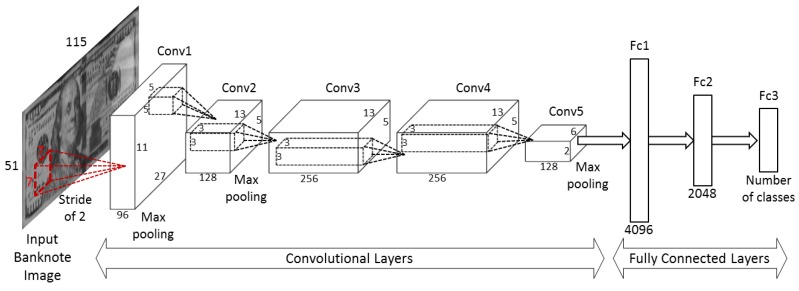
Architecture of the convolutional neural network (CNN).

The CNN used in our proposed method was inspired by the AlexNet architecture [24]. Our network architecture includes five convolutional layers, denoted by Conv1 to Conv5, and three fully-connected layers, denoted by Fc1 to Fc3, as shown in Figure 5. Rectified linear unit (ReLU) layers are adopted in all the convolutional and fully connected layers. This network unit performs a threshold operation that sets all the negative input values of *x* to zero, as shown in Equation (1). The usage of the ReLU active function helps to improve the generalization, simplify the computation, and increase the training speed of the deep network [34]:

(1) $$f(x)=\{\begin{array}{c} x,\;x\geq 0\\ 0,\;x<0 \end{array}$$

In the first two layers of Conv1 and Conv2, cross channel normalization (CCN) and max pooling layers are included [24]. The mathematical equation of CCN is as follows:

(2) $${\bar{a}}_{x,y}^{i}=\frac{a_{x,y}^{i}}{{\left(K+\alpha \sum_{j=\max(0,i-\frac{n}{2})}^{\min(N-1,i+\frac{n}{2})}{\left(a_{x,y}^{j}\right)}^{2}\right)}^{\beta }},$$

In Equation (2), ${\bar{a}}_{x,y}^{i}$ is the value obtained by normalization [24]. In this research, 1, 0.0001, and 0.75 are used for the values of *K*, *α*, and *β*, respectively. $a_{x,y}^{i}$ represents the neuron activity computed by the application of the *i*th kernel at location (*x*, *y*), and executes normalization for the adjacent *n* kernel maps at the identical spatial position [24]. In this research, we set *n* at 5. *N* shows the total number of kernels in the layer.

The max pooling layer is also presented in the last convolutional layer (Conv5) and is followed by three fully connected layers. A dropout layer is inserted in the Fc2 layer to prevent over-fitting in the training process [35].

As explained in previous research [24,35], CNN-based classification methods usually have an over-fitting problem, which can degrade a recognition accuracy with test data, although the accuracy with the training data is still high. To address this issue, we adopted data augmentation and dropout methods [24,35], which can lessen the effects of the over-fitting problem. The detailed explanations of data augmentation are shown in Section 3. The dropout method is a form of regularization technique in NN that randomly disconnects the connections between nodes in the hidden layers of the fully connected network [24,35]. The idea of the technique is the element-wise multiplication of the input vector **x** to the network node with the vector **r** in which each element is a Bernoulli random variable and has a probability *p* of being 1 [35]. The feed-forward operation of network node with dropout is as follows:

*y* = *f* (**w**(**x·r**) + *b*) (3)

where **w** and *b* are the weights and bias at the network node, respectively. With the active function denoted by *f*(·), *y* is the output value of the node. In our study, the dropout layer with zero connection probability of 65% was adopted directly before the 3rd fully connected layer, as shown in Table 2.

The detailed descriptions of the network structure and feature map sizes at each layer are given in Table 2. In Table 2, the size of the feature map in the *i*th convolutional layer (width × height × depth in pixels, denoted by *w_i_* × *h_i_* × *d_i_*, respectively) is calculated by the size of its preceding (*i* − 1)th layer (denoted by *w_i_*_− 1_ × *h_i_*_− 1_ × *d_i_*_− 1_) by the following formulas [26,36]:

(4) $$w_{i}=\frac{w_{i-1}-f+2p}{s}+1,$$

(5) $$h_{i}=\frac{h_{i-1}-f+2p}{s}+1,$$

(6) $$d_{i}=\{\begin{array}{cc} d_{i-1} & \mathrm{if}\;i\;\mathrm{is}\;a\;\mathrm{pooling}\;\mathrm{layer}\\ k & \mathrm{if}\;i\;\mathrm{is}\;a\;\mathrm{convolutional}\;\mathrm{layer} \end{array}$$

where the number of weights per filter is (*f* × *f* × *d_i_*), number of filters is *k*, *s* is the stride number, and *p* is the amount of zero padding. In our proposed method, input banknote images are resized to the same size of 115 × 51 pixels. As shown in Table 2, the feature map size changes at each stage of the convolutional layers and have the size of 6 × 2 × 128 at the final convolutional layer of Conv5, resulting in 1536 banknote image features to be fed into the following fully connected layers.

The CNN consists of two components: convolutional layers and fully connected layers. In the convolutional layers, image feature extraction is done via several filters with different sizes followed by pooling stages; meanwhile, classification is conducted by fully connected layers. In our method, three fully connected layers are used for the classification of banknotes with the number of nodes at each layer shown in Table 2. The number of nodes in the last layer is equal to the number of banknote classes (denomination and input direction of banknotes) to be classified.

In the network training process, filter coefficients in the convolutional layers and weights in the fully connected layers are learned by training banknote image sets. Complete network models with trained coefficient and weights are stored in memory for use in the testing process. In our method, banknotes are pre-classified by size; therefore, the training is performed separately on each size class to learn different network models for classifying type and direction of banknote images belonging to the corresponding size class.

### 2.5. Score Level Fusion and Classification by Softmax Function

In the final step of our proposed method, banknotes are classified according to country of origin, denomination, and input direction by the fully connected layers of the trained CNN models. The input banknote, in our method, is captured images from both sides. If the banknote is presented into the system in the forward direction, the pair of captured images consists of the A and C direction images of the banknote; and if the input direction is backward, the B and D direction images can be captured. Both images are fed separately into the trained NN model corresponding to their pre-classified size class, as shown in Figure 1. The outputs of the fully connected layer of Fc3 are considered as the classification scores of the input banknote images, denoted by *sf_i_* and *sb_i_*, (*i* = 1, …, *N*), which are scores of the front side image and back side image, respectively, and *N* is the number of classes. In general, the output score value, corresponding to the banknote image’s genuine class is higher than those belonging to the other classes. First, we rearrange the order of the scores of the bank side image corresponding to the score indices of the front side image. For instance, the front image is in the A direction, while the back image is in the C direction; at this step, we swap the positions of the two scores corresponding to these two directions. Then, we take the average value of the two image pair scores to obtain the combine score *s_i_* of banknote (SUM rule of score fusion) as the following equation:

(7) $$s_{i}=\frac{1}{2}(sf_{i}+sb_{i}),$$

We consequently apply a normalized exponential function (softmax function) [30] to the combined scores. The softmax function is used in the multiclass classification to not only map a set arbitrary real values to real values in the range (0, 1) that can represent probability, but also help to highlight the largest values among the set and suppress small values [31]. The formula of the softmax function is as follows:

(8) $$p_{i}=\frac{\exp(s_{i})}{\sum_{i=1}^{N}\exp(s_{i})},$$

Among the *N* banknote classes, the one corresponding to the maximum value of *p_i_* (*i* = 1, …, *N*) is assigned as the belonging class of the input banknote image pair.

## 3. Experimental Results

**Figure 6:**
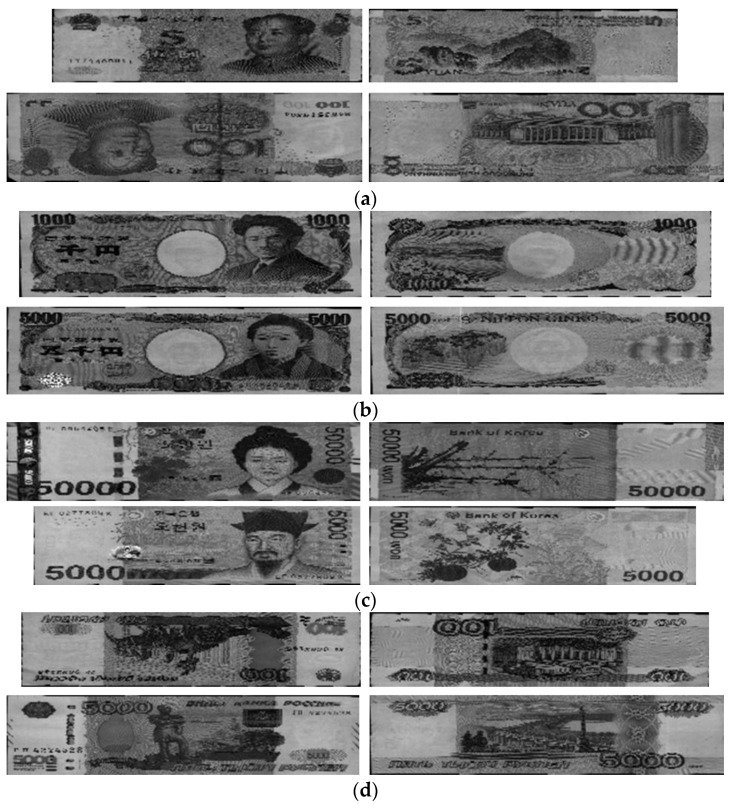

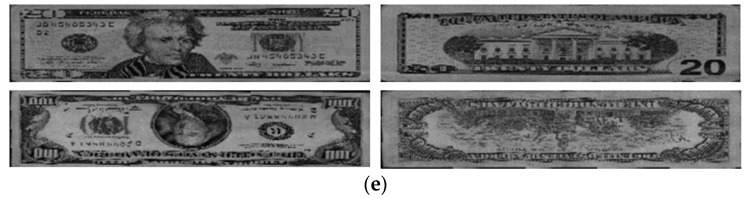
Examples of banknote images used for experiments: (**a**) Chinese yuan (CNY); (**b**) Japanese yen (JPY); (**c**) Korean won (KRW); (**d**) Russian ruble (RUB); and (**e**) United State dollar (USD).

In our study, experiments using the proposed method were conducted on a multi-national banknote image database containing images from six national currencies: CNY, EUR, JPY, KRW, RUB, and USD. A total of 64,668 images were captured from both sides of 32,334 banknotes belonging to 62 currency denominations from six countries. The number of classes is four times the number of denominations because of the inclusion of four directions; therefore, there are 248 classes of banknote to be classified in our study. In Table 3, we give the details, number of images, and classes (denominations and directions) of each country’s banknote in the dataset. In comparison with the multi-national databases used in the previous work, our experimental database contains more numbers of national currencies and denominations than those of the previous studies in [10,11,12], and more number of images than that of [13], as shown in Table 4. Examples of banknote images of CNY, JPY, KRW, RUB, and USD are shown in Figure 6. EUR banknote image examples are given in Figure 3. We made our database of multi-national currencies and trained CNN model public through [37], such that other researchers can compare and evaluate its performance.

**Figure 7:**
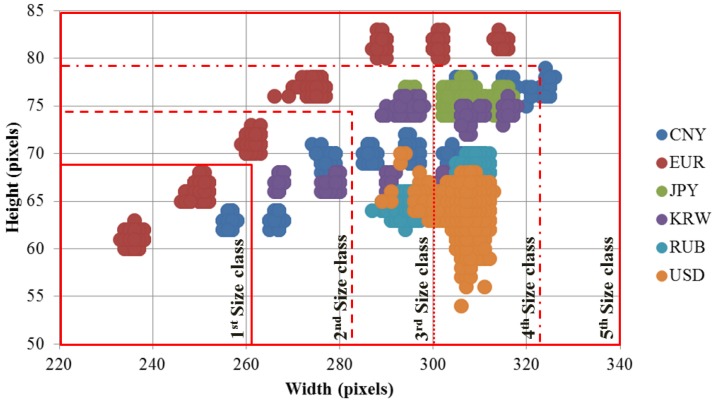
Definition of size classes in ascending order of height and width of banknote.

In the first experiment, we investigated the size information of banknote images for size pre-classification. Based on the size distributions shown in Figure 4, we defined five size classes in an ascending order of heights and widths of banknote region segmented images, considering the separation and overlapping of banknote type sizes. Figure 7 illustrates the definition of size class boundaries defined on size distribution scatter plots. It can be seen from the Figure 7 that the banknote images from national currencies consisting of multiple-size notes such as CNY, EUR, EUR, JPY and KRW can be pre-classified into different size classes. The detail description of banknote classes in each size class is given in Table 5. It can be seen from Table 5 that the third and fourth size classes consist of the most numbers of classes in comparison to the other size classes. The reason is as follows: From Figure 4 and Figure 7, we can see that USD’s size distribution overlaps with several size groups of banknotes from other countries. Because of this fact, we included 68 classes of USD in both the third and fourth size classes, and consequently, the numbers of classes in these two size classes were increased.

The performance of our proposed method was measured by conducting a two-fold cross-validation method. To do so, we randomly divided the dataset into each size class shown in Table 5, into two subsets, one for training and another one for testing, and repeated the processes with alternating these two subsets. In the cases of the CNY, EUR, JPY, and KRW banknotes, we performed data augmentation for expanding and generalizing the datasets because the numbers of images for these four kinds of banknotes are relatively smaller than those for RUB and USD. For data augmentation, we randomly cropped the original image in the dataset in the range of 1~5 pixels on the four boundaries. The numbers of images in CNY, EUR, JPY, and KRW datasets were multiple by the factor of 20, 3, 10 and 24, respectively, for being relatively similar to that of RUB dataset. These augmented data were used for training, and based on this scheme of data augmentation, the unbalance of training dataset of each country’s banknote can be reduced. We also list the number of images in each country’s banknote dataset after performing data augmentation in Table 3. Training and testing experiments were performed by using the MATLAB implementation of CNN [38] on a desktop computer equipped with an Intel^®^ Core™ i7-6700 CPU @ 3.40 GHz [39], 64 GB memory, and an NVIDIA GeForce GTX TITAN X graphics card with 3072 CUDA cores, and 12 GB GDDR5 memory [40].

**Figure 8:**
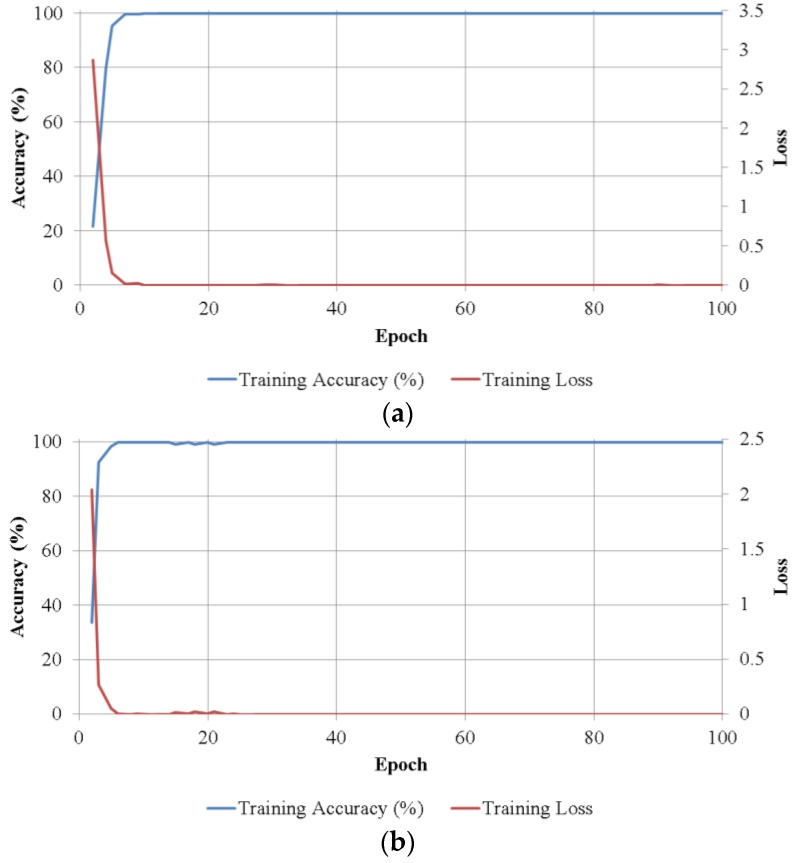

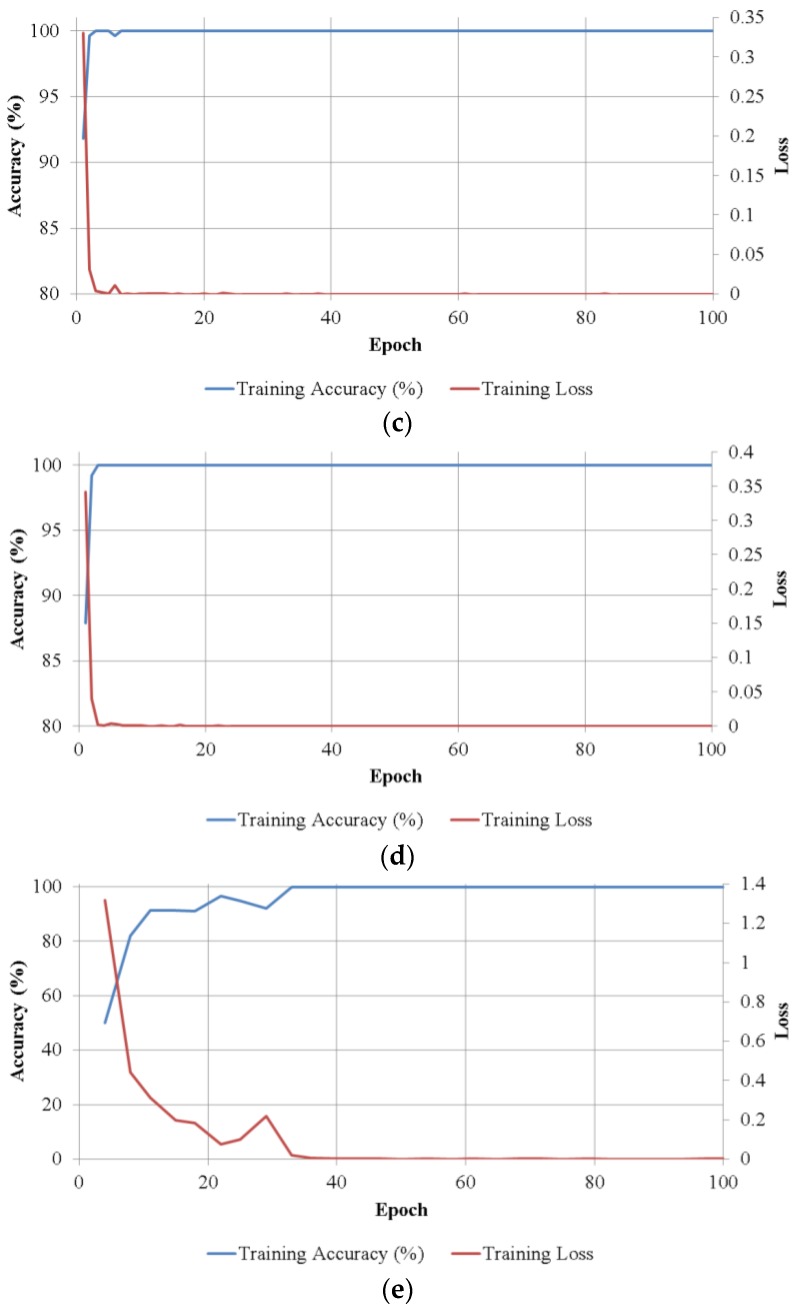
Convergence graphs with average accuracies and losses according to the number of training epochs on two trainings of two-fold cross-validation on the five size classes: (**a**) 1st size class; (**b**) 2nd size class; (**c**) 3rd size class; (**d**) 4th size class; and (**e**) 5th size class.

In the CNN training experiments, we trained five separate network models for classifying banknotes in each of five size classes, and repeated it twice for two-fold cross-validation. The training method used in this research is stochastic gradient descend (SGD), or on-line gradient descent [30], which updates the network weights based on one data point at a time. Network training parameters were selected as follows: the learning process iterated over 100 epochs, with the initial learning rate of 0.01 and reduced by 10% at every 33 epochs; the probability of dropout in the second fully connected layer is set to 65%. Figure 8 shows the training convergence graphs of the average batch loss and classification accuracy values of the two trainings in two-fold cross-validation according to the epoch number on each size class. As shown in Figure 8, in all cases, accuracies increased and loss curve approaches zero with the increment of training epochs.

Figure 9 shows the 96 trained filters in the first convolutional layers of the CNN models obtained by two trainings of two-fold cross-validation on each size class. The original size of each filter is 7 × 7 pixels as shown in Table 2. For visualization purpose, we resized each filter with the factor of 5 and scaled the original real values of filter weights to the range of gray scale images (0 to 255 of unsigned integer type).

With the trained CNN models, we measured the classification accuracies on the multi-national currency datasets. In the first experiment, we conducted the testing process of two-fold cross-validation separately on each dataset. We also compared the performance of the proposed method with that of the method in the previous study [5] using two-fold cross-validation. When using the previous method in [5], classification scores were selected as the matching distances between banknote’s feature vector to the trained K-means centroids of banknote classes [5], and the score fusion method was also the SUM rule as shown in Equation (7). The comparative experimental results of the two-fold testing processes on the five banknote size classes are given in Table 6. The average testing accuracies were calculated based on the number of accurately classified samples on each testing subset of the two-fold cross-validation method as follow [41]:

(9) $$ACC=\frac{GA_{1}+GA_{2}}{N},$$

where *ACC* is the average testing accuracy, *GA*_1_ and *GA*_2_ are the number of correctly recognized samples (genuine acceptance cases of banknotes) in the first and second testing subsets, respectively, and *N* is the total number of the samples in the dataset.

In the final testing experiment, we tested the recognition accuracy of the overall workflow of the proposed method as shown in Figure 1. First, the banknote images in each testing subsets were pre-classified into five size classes according to their size information as shown in Table 5. Banknote features were subsequently extracted and used for classification of national currency, denomination, and input direction by using the corresponding CNN model of the pre-classified size class. These overall testing results are also shown in Table 6. With the average testing accuracy, we evaluated the performance of the proposed method in comparison to the accuracies reported for multi-national banknote recognition methods used in previous works [12,13], as shown in Table 7.

It can be seen from Table 6 that the proposed method correctly classifies banknotes from multiple countries in all test experiment cases. In the cases of the third and fourth size classes, our CNN-based method outperformed the previous study’s method in terms of higher recognition accuracy. From Table 7, we can see that although the number of banknote images in our experimental database were greater than that in other studies of [12,13], and consisted of more classes than that in [12], the proposed CNN-based multi-national banknote classification method outperforms the previous methods [12,13], in term of higher reported recognition accuracies.

**Figure 10:**
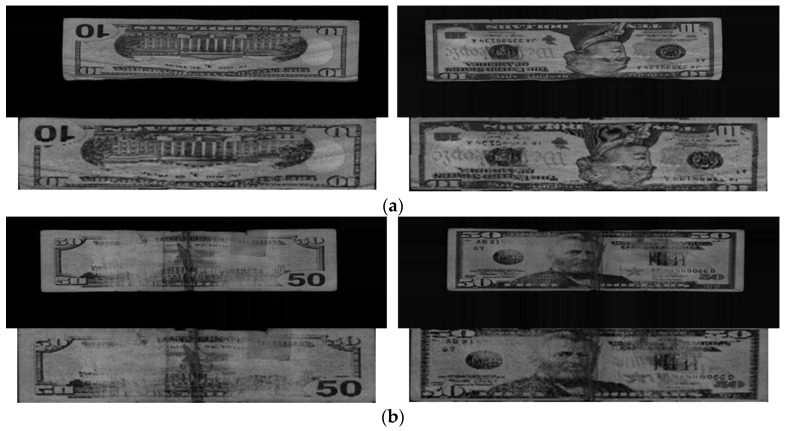
Classification error cases of the testing results when using previous method [5]: (**a**) Case 1; (**b**) Case 2.

Examples of classification error cases when using the previous method are shown in Figure 10, in which both side images of misclassified banknotes were presented with the upper images are the original captured banknote images and the lower images was banknote region segmented image of the upper one.

It can be seen from Figure 10 that Case 1 consists of banknote images captured from a creased banknote that caused a loss of information when performing sub-sampling on images in the method from [5]; and Case 2 was from a severely bleached and damaged banknote. Both cases are from USD dataset which is included in the third and fourth size classes. The physical damage on these banknotes caused the misclassification when using the previous method in [5]. However, when using CNN, all the cases were correctly recognized, due to the robustness of CNN to the quality of captured images [26].

## 4. Conclusions

In this research, we proposed a multi-national banknote recognition method based on the adoption of CNN for feature extraction and classification. Banknote images are normalized to have the same size, and fed into trained CNN models corresponding to the pre-classified size classes. When passing through the neural network, banknote features are extracted by the convolutional layers and classified into the national currency type, denomination, and input direction by the fully connected layers of the network. Our experimental results using two-fold cross-validation on the multi-national currency dataset show that the proposed CNN-based banknote recognition method yields better accuracies than the method in the previous study.

Although CNN-based classification has been used in various fields due to its high performance, it has the disadvantage of requiring intensive training with a lot of training data. However, it is often the case to have difficulty in collecting a lot of training data in actual experimental environments. Therefore, the procedure of increasing data by data augmentation is performed. In order to reduce this disadvantage, we made our trained CNN model with collected database of multi-national currencies public through [37], such that other researchers can easily compare and evaluate its performance.

In future work, we plan to combine the proposed recognition method with the classification of fitness for recirculation of banknotes for rejecting damaged or stained banknotes not suitable for using in practice. We also intend to further study about the application of CNN to other problems related to banknote classification, such as counterfeit detection and serial number recognition.

## Figures and Tables

**Figure 9:**
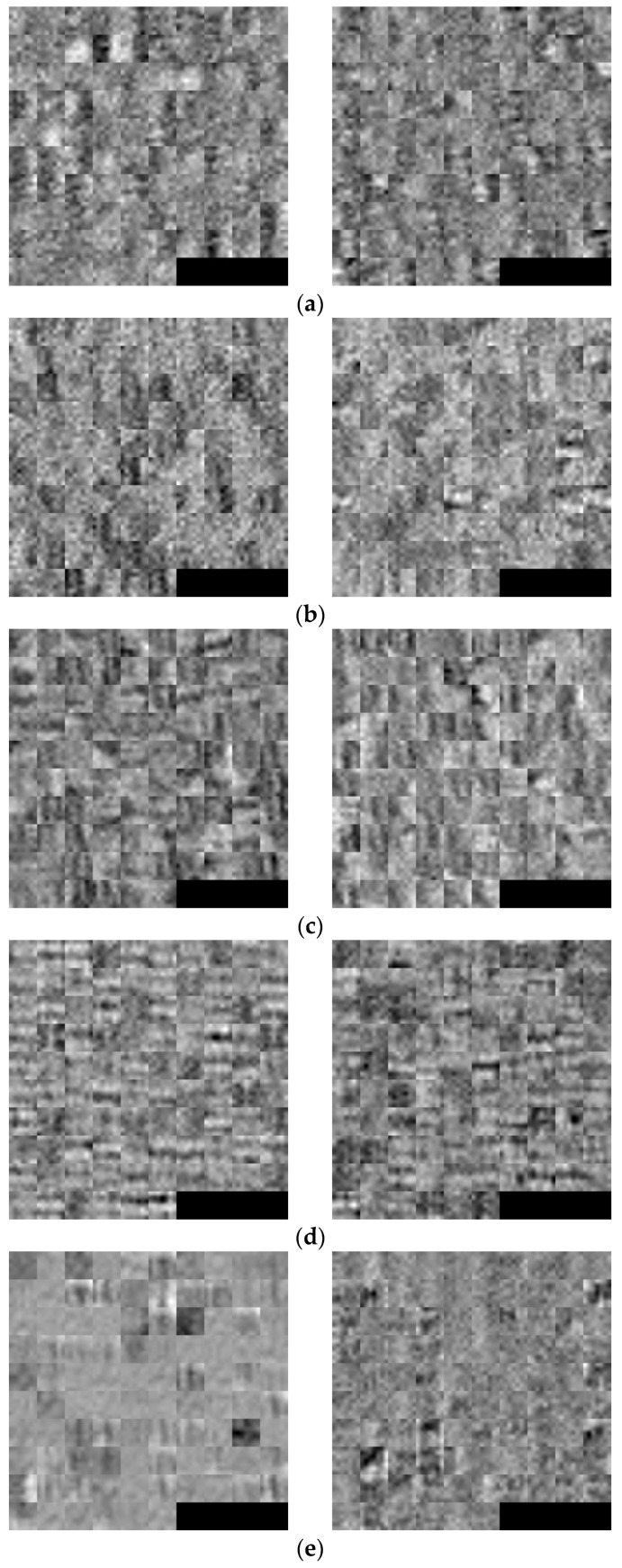
Visualization of filter weights in the first convolutional layers of the CNN model in each size class, in which the left and right images are from the training results on the first and second subsets for two-fold cross-validation, respectively: (**a**) 1st size class; (**b**) 2nd size class; (**c**) 3rd size class; (**d**) 4th size class ; and (**e**) 5th size class.

**Table 1:** Comparison of proposed method and related works on multi-national banknote recognition.

Category	Method	Strength	Weakness
Separate recognition of multiple currencies	Using QWT and GGD for feature extraction and NN for classification [4]. Using similarity map, PCA and K-means-based classifier [5]. Using HSV color features and Euclidean distance-based classifier [6].	Advantage in resource usage and processing time, as well as classifier complexity due to lower numbers of banknote classes to be classified.	Type of currency to be classified needs to be manually selected.
Simultaneous recognition of multiple currencies	Using NN [8]. Using SIFT descriptors with various classifiers (SVM, ANN, HMM) for comparison [9]. Using multi-template correlation matching [10]. Using LDA classification method on EHD features [11]. Using GA and NN [12]. Using HMM on banknote texture characteristics [13]	Manual selection of currency type to be classified is not necessary.	As the number of classes to be classified increases with the number of currencies, the classification efficiency tends to decrease.
	Using CNN (**Proposed method**)	Through intensive training of CNN, the classification performance is high irrespective of the variety of multi-national currency images.	Time consuming procedure for CNN training is required.

**Table 2:** Structure of the CNN using in our banknote recognition method.

Layer Type		Number of Filters	Size of Kernel	Number of Stride	Padding	Size of Feature Map
Image Input Layer					115 × 51 × 1
Conv1	Convolutional Layer	96	[7 7]	[[2 2]	[0 0]	55 × 23 × 96
	ReLU Layer					
	CCN Layer					
	Max Pooling Layer	1	[3 3]	[2 2]	[0 0]	27 × 11 × 96
Conv2	Convolutional Layer	128	[5 5]	[1 1]	[2 2]	27 × 11 × 128
	ReLU Layer					
	CCN Layer					
	Max Pooling Layer	1	[3 3]	[2 2]	[0 0]	13 × 5 × 128
Conv3	Convolutional Layer	256	[3 3]	[1 1]	[1 1]	13 × 5 × 256
	ReLU Layer					
Conv4	Convolutional Layer	256	[3 3]	[1 1]	[1 1]	13 × 5 × 256
	ReLU Layer					
Conv5	Convolutional Layer	128	[3 3]	[1 1]	[1 1]	13 × 5 × 128
	ReLU Layer					
	Max Pooling Layer	1	[3 3]	[2 2]	[0 0]	6 × 2 × 128
Fc1	Fully Connected Layer					4096
	ReLU Layer					
Fc2	Fully Connected Layer					2048
	ReLU Layer					
	Dropout Layer					
Fc3	Fully Connected Layer					Number of Classes
	Softmax Layer					

**Table 3:** Number of images and classes in the experimental multi-national banknote database.

Currency	Number of Images	Number of Images after Data Augmentation	Number of Classes
CNY	626	12,520	40
EUR	4324	12,972	44
JPY	1462	14,620	28
KRW	536	12,864	28
RUB	12,146	12,146	40
USD	45,574	45,574	68

**Table 4:** Comparison of the numbers of images and classes in the experimental databases used in previous studies and in this study.

Study	Number of Images	Number of National Currencies	Number of Denominations
[10]	100,797	5	55
[11]	14,000	4	14
[12]	4025	4	23
[13]	105	23	101
This study	64,668	6	62

**Table 5:** Number of banknote classes in each size classes (*h* and *w* stand for height and width in pixel units of segmented banknote images).

Size Class	Size Range	Number of Images	Number of Classes
1st Size Class	*h* < 69 and *w* < 261	2146	20
2nd Size Class	69 ≤ *h* < 74 and 261 ≤ *w* < 283	952	24
3rd Size Class	74 ≤ *h* < 79 and 283 ≤ *w* < 300	55,032	128
4th Size Class	74 ≤ *h* < 79 and 300 ≤ *w* < 322	51,500	136
5th Size Class	Remaining banknote size	1044	16

**Table 6:** Testing results of the proposed method in comparison with those of the previous study on separated size classes (unit: %). 1st Testing Accuracy and 2nd Testing Accuracy mean the accuracies of the testing on the 1st and 2nd subsets of banknote images in the two-fold cross-validation method, respectively.

Size Class	Previous Method [5]			Proposed Method		
	1st Testing Accuracy	2nd Testing Accuracy	Average Testing Accuracy	1st Testing Accuracy	2nd Testing Accuracy	Average Testing Accuracy
1st Size Class	100	100	100	100	100	100
2nd Size Class	100	100	100	100	100	100
3rd Size Class	99.964	99.949	99.956	100	100	100
4th Size Class	99.984	99.961	99.973	100	100	100
5th Size Class	100	100	100	100	100	100
Whole dataset (with size pre-classification)	99.986	99.969	99.978	100	100	100

**Table 7:** Comparison of recognition accuracy of the proposed method and previous studies on multi-national banknote classification.

Recognition Method	Multi-National Banknote Image Dataset			Error Rate (%)	Rejection Rate (%)
	Number of Image	Number of National Currency	Number of Denomination		
[12]	4025	4	23	0	3
[13]	105	23	101	0	2
Proposed method	64,668	6	62	0	0
